# Th1 Cytokines Inhibit Acinar Morphogenesis and Milk Protein Expression in 3D Mammary Cultures

**DOI:** 10.3390/biomedicines13061455

**Published:** 2025-06-12

**Authors:** Lih-Ju Chen, Yi-An Su, Ting-Hui Lin, Wan-Ting Liao, Chun-Chi Wu, Chen-Chu Lin, Chang-Han Chen, Tsai-Ching Hsu, Ya-Wen Yang, Yi-Ju Lee

**Affiliations:** 1 Department of Post-Baccalaureate Medicine, College of Medicine, National Chung Hsing University, Taichung 402, Taiwan; 2 Division of Neonatology, Changhua Christian Children’s Hospital, Changhua 500, Taiwan; 3 Institute of Medicine, Chung Shan Medical University, Taichung 402, Taiwan; 4 Department of Biomedical Sciences, Chung Shan Medical University, Taichung 402, Taiwan; 5 Department of Chinese Medicine, Show Chwan Memorial Hospital, Changhua 500, Taiwan; 6 Department of Health Industry Technology Management, Chung Shan Medical University, Taichung 402, Taiwan; 7 Department of Medical Research, Taichung Veterans General Hospital, Taichung 407, Taiwan; 8 Department of Applied Chemistry, and Graduate Institute of Biomedicine and Biomedical Technology, National Chi Nan University, Nantou 545, Taiwan; 9 Immunology Research Center, Chung Shan Medical University, Taichung 402, Taiwan; 10 Department of Nutrition, Show Chwan Memorial Hospital, Changhua 500, Taiwan

**Keywords:** IFN-γ, TNF-α, mammary gland, 3D culture, morphology, lactation

## Abstract

**Background**: The principal function of mammary glands is to produce milk to nourish the newborn. Optimal lactation is controlled by various hormones, growth factors, and cytokines. **Objectives**: Using 3D cultures of primary mouse mammary epithelial cells, we explored the effects of T helper (Th)1 cytokines, interferon (IFN)-γ, and tumor necrosis factor (TNF)-α on the structure and function of mammary cells as well as the underlying mechanism. **Methods**: Three-dimensional cultures of mammary cells were treated with IFN-γ/TNF-α, and milk protein expression and acinar structures were analyzed by immunoblotting and immunofluorescence microscopy. **Results**: Our results revealed that combined treatment with IFN-γ and TNF-α inhibits prolactin-induced STAT5 tyrosine phosphorylation and β-casein expression. These cytokines also disrupted the structure of mammary acini, resulting in smaller or no lumens, disordered cell arrangements, and multilayered cells in certain regions. Additionally, some cells became elongated rather than maintaining their usual cube-like shape. Since cell proliferation and death can modulate the structural organization of acini, we examined the influences of IFN-γ and TNF-α on these events. Combined cytokine treatment moderately increased cell proliferation and cell death. Notably, stimulation with IFN-γ and TNF-α induced the expression of inducible nitric oxide synthase (iNOS), and the inhibition of iNOS partially restored acinar morphology and β-casein expression, revealing a novel mechanism for cytokine-induced acinar disruption. **Conclusions**: When a Th1 cytokine milieu is dominant, such as during inflammation and infection, IFN-γ and TNF-α might cause mammary gland ductal occlusion and lactation insufficiency.

## 1. Introduction

Mammary gland development primarily occurs in adulthood. At the onset of puberty, the rudimentary duct elongates and branches to form a ductal tree that extends through the fat pad. During pregnancy, alveolar buds are generated at the ends of these branches, which then undergo maturation and functional differentiation in preparation for subsequent lactation. Lactation is the functional phase of mammary gland development. Copious milk is synthesized and secreted to nourish the newborn [1]. At weaning, the mammary gland is remodeled to a pre-pregnant state via the massive death of secretory alveolar cells, a process termed involution [2]. The complex program of mammary gland development involves cell proliferation, differentiation, and death and is regulated by systemic and local factors, such as hormones, growth factors, and cytokines [1,3].

The mammary gland is an epithelial organ. Two types of epithelia, luminal and basal cells, generate a bi-layered structural network that is essential for mammary function. Luminal cells form the ducts and alveoli, while basal cells, also known as myoepithelial cells, are positioned along the basal surface of the luminal cells. During lactation, luminal cells within the alveoli synthesize and secrete milk. Contraction of myoepithelial cells helps to eject and transport milk through the ductal system, which ultimately drains into openings in the nipple. A basement membrane lies between the mammary epithelium and the surrounding stroma, serving as a physical and functional boundary [4]. The stroma contains a variety of cells, including adipocytes, fibroblasts, vascular endothelial cells, and immune cells, all of which support the structure and function of mammary glands [4,5,6].

Immune cells are not confined to the stroma. Some, such as macrophages and T cells, are closely associated with epithelial cells [5,7,8,9,10]. It has been recently reported that a distinct population of mammary intraepithelial lymphocytes appears during pregnancy [11]. Although not the most abundant leukocytes, CD4^+^ T helper (Th) cells are present in the mammary gland, and their numbers increase during lactation [12]. CD4^+^ Th cells are classified into several lineages, including Th1, Th2, Th17, follicular helper T (Tfh), and regulatory T (Treg) cells. Each lineage produces its own signature cytokines and performs specific functions. Among them, Th1 cells secrete interferon (IFN)-γ and tumor necrosis factor (TNF)-α, which activate macrophages and protect against intracellular pathogens. Th2 cells secrete interleukin (IL)-4, IL-5, and IL-13, which mediate the defense against extracellular parasites and the development of allergic reactions [13,14]. Overall, the immune system plays pivotal roles in mammary gland development and immunosurveillance [6].

The roles of Th1 and Th2 cytokines in mammary gland development have been previously documented. Th2 cytokines, particularly IL-4 and IL-13, promote alveologenesis during pregnancy [15]. In contrast, Th1 cells interact with CD11c^+^ antigen-presenting cells to inhibit branching morphogenesis during puberty. This is mediated by their secretion of IFN-γ [7]. However, the impact of Th1 cytokines on lactation remains unclear. Given that infection or inflammation could tip the Th1/Th2 balance toward a Th1-dominant cytokine milieu with elevated levels of IFN-γ and TNF-α, we sought to determine whether this could jeopardize mammary gland development and lead to lactation failure. Mastitis is an inflammation of the mammary glands that commonly occurs during lactation, mainly due to bacterial infection or milk stasis. It is associated with increased levels of inflammatory mediators, accompanied by decreased milk production [16,17]. Interestingly, a recent study has shown that women with subclinical mastitis exhibit a predominant Th1/proinflammatory cytokine profile, suggesting a potential link between Th1 cytokines and mammary gland dysfunction [18].

IFN-γ acts on numerous cell types, including immune cells and non-immune cells, as the IFN-γ receptor is ubiquitously expressed. Through the activation of the JAK-STAT1 pathway, IFN-γ promotes host defense and tumor immunosurveillance and regulates tissue remodeling [19,20]. TNF-α also functions as a pleiotropic cytokine. Signaling through complex I activates NF-κB and MAPK, promoting inflammation, cell survival, and proliferation. Alternative signaling via complex II results in apoptosis and necroptosis [21].

In this study, we employed a previously established 3D culture system in which primary mouse mammary epithelial cells are cultured on a reconstituted basement membrane matrix (Matrigel). This model is considered physiologically relevant, as it can recapitulate acinar architecture and lactogenic function in vitro [22]. With this culture system, we demonstrated that the Th2 cytokines IL-4 and IL-13 stimulate β-casein expression. These cytokines also promote cell proliferation, leading to acinus enlargement [23]. These results are consistent with their positive impacts on alveologenesis during pregnancy [15]. In light of the opposing effects of Th1 and Th2 cytokines on immune responses and the observation that Th1 cells negatively regulate organogenesis via IFN-γ, we investigated the influence of the Th1 cytokines IFN-γ and TNF-α on the structure and function of mammary cells [7,24]. We found that combined treatment with IFN-γ and TNF-α inhibits prolactin-induced β-casein expression and disrupts acinar morphology. Notably, inducible nitric oxide synthase (iNOS) was identified as a potential mediator of these detrimental effects. These findings provide new mechanistic insights into how inflammatory cytokines contribute to lactation failure under conditions such as mastitis.

## 2. Materials and Methods

### 2.1. Reagents

Recombinant murine IFN-γ and TNF-α were purchased from PeproTech (Rocky Hill, NJ, USA). The NOS inhibitor L-NAME was supplied by Cayman Chemical (Ann Arbor, MI, USA).

### 2.2. Cell Culture

All experiments were conducted with second-passage mammary epithelial cells. Mammary alveoli were isolated from mid-pregnant ICR mice as previously described [25]. Briefly, mammary glands from 5~6 mice were digested with a solution containing collagenase A (3 mg/mL) and trypsin (1.5 mg/mL) at 37 °C and subjected to differential centrifugation (107× *g*, 375× *g*) to enrich the epithelial cells. Following washing and centrifugation, the cells were pooled and plated onto Matrigel (Corning, Bedford, MA, USA) in nutrient mixture F-12 (Sigma-Aldrich, St. Louis, MO, USA) containing 10% fetal bovine serum (Gibco, Carlsbad, CA, USA), 1 mg/mL of fetuin (Sigma-Aldrich), 5 ng/mL of EGF (Sigma-Aldrich), 5 μg/mL of insulin (Sigma-Aldrich), and 1 μg/mL of hydrocortisone (Sigma-Aldrich). After 72 h, the cells were trypsinized and cultured onto Matrigel overnight. The culture medium was then changed to Dulbecco’s modified Eagle’s medium (DMEM)/nutrient mixture F-12 (Gibco) containing hydrocortisone and insulin, and the cells were stimulated with IFN-γ (10 ng/mL), TNF-α (10 ng/mL), or prolactin (3 μg/mL). The concentrations of IFN-γ and TNF-α were based on studies for the examination of milk synthesis and barrier function in mammary epithelial cells [26,27]. In most experiments, the cells were pretreated with cytokines for 1 h before exposure to prolactin for 2 days. This pretreatment period was selected to ensure effective inhibition, as cytokine signaling activity peaks within this time frame [7]. A 2-day prolactin stimulation period was chosen to elicit the maximal induction of β-casein expression [28]. To examine the involvement of iNOS in the inhibitory effects of IFN-γ and TNF-α on mammary structure and function, the iNOS inhibitor L-NAME was used at a concentration of 1 mM, as reported previously, and was co-administered with cytokines prior to prolactin stimulation [29]. Of note, all experiments were repeated at least three times using independently isolated primary cell preparations (biological replicates). In this study, animals were obtained, maintained, and used in accordance with the policies of the Institutional Animal Care and Use Committee of the Chung Shan Medical University (IACUC Approval No. 2228).

### 2.3. Immunoprecipitation and Western Blot Analysis

Cells were lysed in lysis buffer containing 50 mM Tris (pH 7.4), 150 mM NaCl, 2 mM EDTA, 1% Triton X-100, 1 mM Na_3_VO_4_, 10 mM NaF, and 1× protease inhibitor cocktail (MedChem Express, Monmouth Junction, NJ, USA). Whole cell lysates were incubated with 1–2 µg of antibody and 30–50 µL of protein A-Sepharose beads (Invitrogen, Rockford, IL, USA) at 4 °C for 4–6 h. Immunoprecipitates or whole cell lysates were subjected to SDS-PAGE, transferred to polyvinylidene fluoride (PVDF) membranes, and probed with antibodies (Table 1). Proteins were visualized using an ECL kit (Millipore, Billerica, MA, USA). Actin served as a loading control. ImageJ version 1.53e (NIH, Bethesda, MD, USA) was used to quantify band intensity.

### 2.4. Immunofluorescence Microscopy

Mammary acini cultured on Matrigel-coated coverslips were fixed in 4% paraformaldehyde for 15 min, permeabilized with 0.5% Triton X-100 for 15 min, and blocked with 1% goat serum for 30 min. F-actin was detected by incubating cells with rhodamine phalloidin (5 U/mL; Invitrogen, Eugene, OR, USA) at room temperature for 20 min, and nuclei were stained with Hoechst 33342 (5 μg/mL; Life Technologies, Carlsbad, CA, USA) for 10 min. For antibody staining, cells were fixed in 4% paraformaldehyde, permeabilized with 100% methanol for 3 min, and then blocked with 1% goat serum for 1 h. Cells were firstly incubated with ZO-1 antibody at 4 °C overnight, followed by Alexa Fluor 546-conjugated anti-rabbit antibody (Life Technologies, Eugene, OR, USA) at room temperature for 2 h. Next, cells were incubated with β-catenin antibody at room temperature for 1 h, then with Alexa Fluor 488-conjugated anti-mouse antibody (Life Technologies) for 1.5 h. They were finally mounted in a DAPI-containing mounting medium (SouthernBiotech, Birmingham, AL, USA). Immunofluorescence of the mammary acini was observed under a Zeiss LSM 510 META confocal laser scanning microscope (Carl Zeiss MicroImaging GmbH, Jena, Germany).

### 2.5. Cell Proliferation Assay

Mammary acini cultured on Matrigel-coated coverslips were stimulated with prolactin or cytokines for 2 days. Before harvesting, the cells were pulsed with 10 μM 5-ethynyl-2′-deoxyuridine (EdU) for 8 h. The cells were then fixed in 4% paraformaldehyde and permeabilized by 0.5% Triton X-100. EdU incorporation was detected using the Click-iT^TM^ Plus EdU Alexa Fluor^TM^ 488 Imaging Kit (Cat# 10637, Life Technologies, Carlsbad, CA, USA). Immunofluorescence was observed under a confocal laser scanning microscope (Zeiss LSM 510 META).

### 2.6. Live/Dead Cell Analysis

Live and dead cells were detected using the LIVE/DEAD Viability/Cytotoxicity Kit (Invitrogen, Eugene, OR, USA) according to the manufacturer’s instructions. Briefly, mammary acini cultured on Matrigel-coated coverslips were stimulated with prolactin and/or cytokines for 2 days. The cells were then washed with PBS and incubated at 37 °C for 15 min with a mixture of 8 μM calcein AM and 10 μM ethidium homodimer-1. The cells were observed under a fluorescence microscope (Zeiss Axio Imager A2).

### 2.7. Statistical Analysis

Data are expressed as mean ± S.E.M. from at least three independent experiments. One-way analysis of variance (ANOVA), followed by Tukey’s multiple comparisons test, was used to assess statistical significance unless otherwise specified. For group comparisons, two-way ANOVA, followed by the Bonferroni post hoc test, was performed. *p* < 0.05 was considered statistically significant. All statistical analyses were performed using GraphPad Prism 5 software (GraphPad Software, San Diego, CA, USA).

## 3. Results

### 3.1. Combined Treatment with IFN-γ and TNF-α Inhibits Prolactin Signaling and Subsequent β-Casein Expression in Primary Mouse Mammary Epithelial Cells

**Figure 1:**
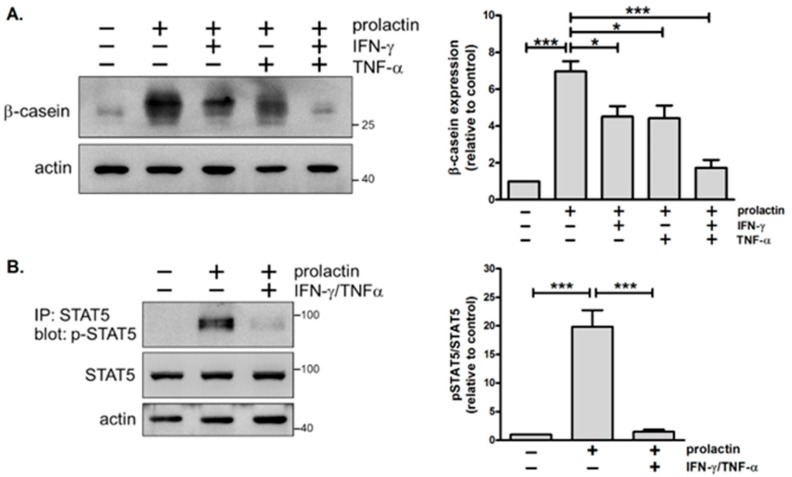
Combined treatment with IFN-γ and TNF-α inhibits prolactin-stimulated STAT5 tyrosine phosphorylation and β-casein expression in mammary cells. (**A**) Immunoblot analysis of β-casein expression in mammary cells pretreated with IFN-γ (10 ng/mL) or TNF-α (10 ng/mL) for 1 h, followed by stimulation with prolactin (3 μg/mL) for 2 days. Actin was used as a loading control (*n* = 4). (**B**) Immunoblot analysis of phospho-STAT5 (p-STAT5) and total STAT5 in mammary cells pretreated with IFN-γ and TNF-α for 1 day, followed by stimulation with prolactin for 15 min. STAT5 immunoprecipitates and total cell lysates were subjected to immunoblotting using antibodies against p-STAT5 and STAT5, respectively (*n* = 3). Data are expressed as fold change relative to the control. * *p* < 0.05, *** *p* < 0.005.

Th1 cells have been shown to hamper the ductal growth and branching morphogenesis of mammary glands via IFN-γ during puberty [7]. Here, we examined the possibility that IFN-γ and another Th1 cytokine, TNF-α, exert negative impacts on lactation. Primary mouse mammary epithelial cells isolated from mid-pregnant mice were cultured on a basement membrane-like matrix (Matrigel), and induction of the milk protein β-casein by prolactin was evaluated. Our results showed that IFN-γ or TNF-α alone inhibits prolactin-stimulated β-casein expression by approximately 40%, and concurrent treatment with both cytokines leads to much greater inhibition (Figure 1A). We thus used a combination of IFN-γ and TNF-α for the following experiments.

Prolactin signals through the prolactin receptor, resulting in activation of the JAK2-STAT5 pathway. Activated STAT5 binds to the promoter of the β-casein gene and induces gene transcription. Given that IFN-γ and TNF-α suppress prolactin-induced β-casein expression, we next examined if they block prolactin signaling. Pretreatment with IFN-γ and TNF-α for 1 day followed by the stimulation of prolactin led to a substantial decrease in levels of STAT5 tyrosine phosphorylation (Figure 1B). Thus, IFN-γ and TNF-α inhibit prolactin signaling and subsequent β-casein expression in mammary cells. This suggests that Th1 cytokines have detrimental effects on lactation.

### 3.2. Combined Treatment with IFN-γ and TNF-α Alters Acinar Morphology

**Figure 2:**
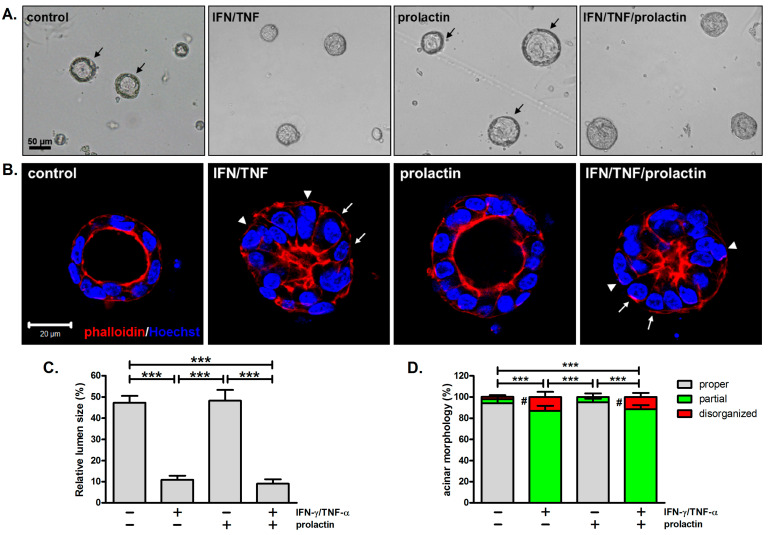
Combined treatment with IFN-γ and TNF-α alters acinar structures. (**A**) Bright-field microscopy images of mammary acini pretreated with IFN-γ/TNF-α for 1 h, followed by prolactin stimulation for 2 days. Arrows indicate outer rings in acini (*n* = 3). Scale bar, 50 μm. (**B**) Confocal images of acini stained with rhodamine phalloidin (red) and Hoechst 33342 (blue). Arrows indicate cells displaying a columnar shape; arrowheads indicate regions with more than one cell layer (*n* = 3). Scale bar, 20 μm. (**C**) Relative lumen size in (**B**) was determined by measuring the cross-sectional area of the lumen and normalizing it to the cross-sectional area of the acinus. *** *p* < 0.005. (**D**) Quantification of the proportions of acini exhibiting a proper, partially retained, and disorganized acinar structure. *** *p* < 0.005, indicating significant differences between treatment groups in terms of proper and partially retained structures. A statistically significant difference in disorganized structures compared to prolactin treatment is denoted by # (*p* < 0.05).

In addition to inhibiting β-casein expression, combined treatment with IFN-γ and TNF-α alters acinar morphology. Untreated acini displayed outer rings, indicative of a single layer of epithelial cells enclosing a hollow lumen (Figure 2A, arrows). Similar structures were observed in prolactin-treated acini, which were enlarged compared to untreated ones (Figure 2A). However, outer rings were not evident in acini treated with IFN-γ/TNF-α, regardless of the presence of prolactin, suggesting that these cytokines alter the normal acinar architecture (Figure 2A).

To further examine the structures of mammary acini, cells were stained with Hoechst 33342 and rhodamine phalloidin to localize the nuclei and F-actin, respectively. For untreated and prolactin-treated acini, a polarized organization was observed, which was composed of a single layer of cells surrounding a lumen, with strong staining of F-actin on the apical surface (Figure 2B). This resembles the alveoli of mammary glands in vivo. In response to IFN-γ and TNF-α, the acini partially retained the alveolar architecture but with smaller lumens (Figure 2B). Lumen size was determined by measuring its cross-sectional area. Because lumen size varies with alveolar size, it was normalized to the alveolar cross-sectional area. Cytokine treatment significantly reduced lumen size to ~20% of the size observed in the control or prolactin-treated acini (Figure 2C). A series of photos taken along the Z-axis of acini also show differences in lumen size among these samples (Supplementary Data, Figure S1). As revealed by nuclear staining, cells within cytokine-treated acini were irregularly arranged, with more than one layer of cells in certain regions (Figure 2B, arrowheads). Additionally, while untreated and prolactin-treated cells exhibited a cuboidal shape, some cells within the IFN-γ/TNF-α-treated acini exhibited a columnar morphology (Figure 2B, arrows). More disorganized structures with no lumens were also observed after cytokine treatment (Supplementary Data, Figure S2). The majority of acini, either untreated or treated with prolactin, exhibited proper acinar morphology. In contrast, most acini exposed to cytokines, with or without prolactin, only partially retained a proper acinar structure (Figure 2D).

### 3.3. Apico-Lateral Polarity Is Largely Preserved in IFN-γ/TNF-α-Treated Acini

**Figure 3:**
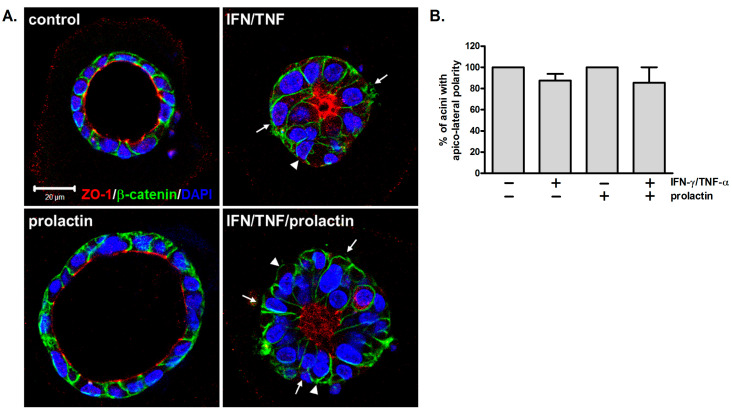
Apico-lateral polarity is largely preserved in IFN-γ/TNF-α-treated acini. (**A**) Confocal images of mammary acini stained with antibodies against ZO-1 (red) and β-catenin (green) and counterstained with DAPI (blue). Arrows indicate cells displaying a columnar shape; arrowheads indicate regions with more than one cell layer (*n* = 3). Scale bar, 20 μm. (**B**) Quantification of the proportion of acini in (**A**) exhibiting proper apico-lateral polarity.

Maintaining tissue polarity is essential for normal functions. In mammary glands, proper tissue polarity ensures the apical secretion of milk into the alveolar lumen and its subsequent transport through ducts. To examine whether concurrent treatment with IFN-γ and TNF-α affects cell polarity, cells were stained with antibodies against ZO-1 and β-catenin to specify the apical and lateral surfaces. Untreated and prolactin-treated acini displayed apical ZO-1 and lateral β-catenin as well as large lumens (Figure 3A). In the cytokine-treated acini, β-catenin was laterally localized and ZO-1 was clustered in the center of the acini, a pattern that coincided with their small or collapsed lumens, suggesting that apico-lateral polarity was roughly preserved (Figure 3A). Similar to the results in Figure 2B, cytokine treatment caused a decrease in lumen size. Moreover, columnar cells (Figure 3A, arrows) and multiple layers of cells were observed (Figure 3A, arrowheads). The results of quantification showed that a substantial proportion of cytokine-treated acini maintained apico-lateral polarity (Figure 3B), consistent with the observations of partial retention of their overall structure (Figure 2B,D).

### 3.4. Combined Treatment with IFN-γ and TNF-α Moderately Enhances Cell Proliferation and Cell Death

**Figure 4:**
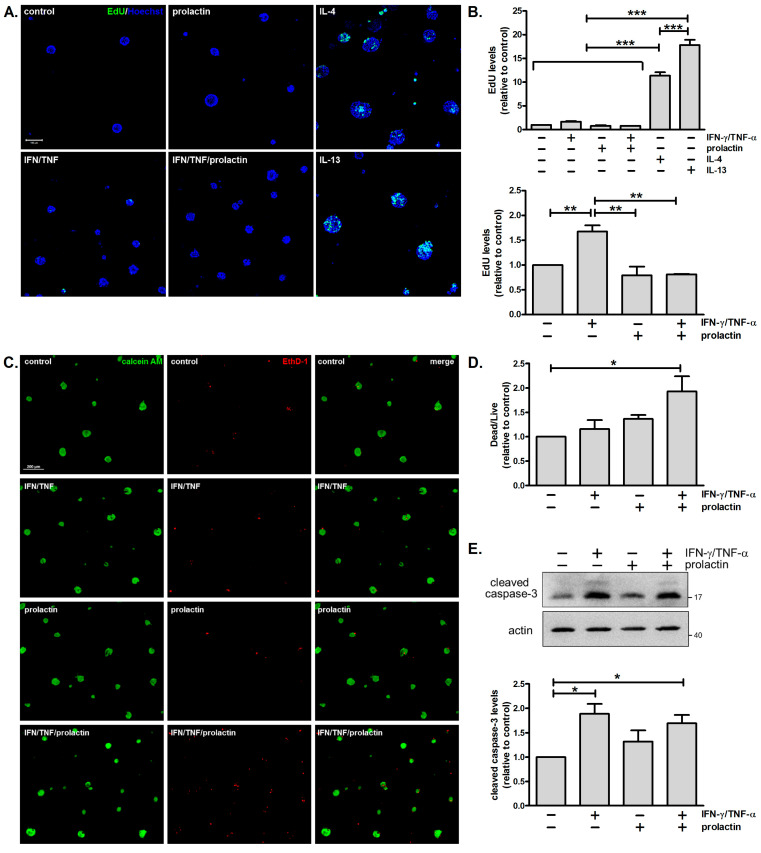
Combined treatment with IFN-γ and TNF-α moderately enhances cell proliferation and cell death. (**A**) Confocal images of mammary acini pulsed with EdU (10 μM; green) for 8 h prior to harvesting. Nuclei were counterstained with Hoechst 33342 (blue). Cells stimulated with IL-4 (10 ng/mL) or IL-13 (10 ng/mL) for 2 days were used as positive controls. Scale bar, 100 μm. (**B**) Quantification of EdU levels in (**A**), normalized to Hoechst 33342 staining and expressed as fold change relative to the control (*n* = 3). ** *p* < 0.01, *** *p* < 0.005. (**C**) Fluorescence microscopy images of acini stained with calcein AM (green) to identify live cells and ethidium homodimer-1 (red) to identify dead cells. Scale bar, 200 μm. (**D**) Quantification of the dead/live cell ratio in (**C**), expressed as fold change relative to the control (*n* = 3). * *p* < 0.05. (**E**) Immunoblot analysis and quantification of levels of cleaved caspase-3 (*n* = 3). * *p* < 0.05.

Failure to maintain proper acinar morphology might be ascribed to dysregulated cell proliferation and/or cell death. Enhanced cell proliferation can cause luminal filling and the formation of multiple layers of cells, whereas excess cell death can lead to reduced acinus size and a disintegrated structure. Given that combined treatment with IFN-γ and TNF-α causes aberrant morphology, we examined whether IFN-γ/TNF-α modulates cell proliferation and cell death. Minimal incorporation of EdU, a nucleoside analog of thymidine, was detected in untreated cells. Combined treatment with IFN-γ and TNF-α moderately enhanced cell proliferation by 1.7-fold, while prolactin or prolactin/IFN-γ/TNF-α had negligible effects. IL-4 and IL-13, which were used as positive controls, substantially stimulated cell proliferation (Figure 4A) [23]. Analysis of all six treatment groups showed significant differences between the IL-4 and IL-13 positive controls and the other treatment groups. Given that IL-4 and IL-13 exerted much stronger pro-proliferative effects, a separate statistical analysis was performed excluding these two groups. This analysis showed that IFN-γ/TNF-α significantly increased cell proliferation (Figure 4B).

To monitor cell death, calcein AM and ethidium homodimer-1 were used to detect live and dead cells, respectively. Ethidium homodimer-1 binds to nucleic acids in cells with damaged membranes, generating red fluorescence. As shown in Figure 4C,D, cell death was more prominent in cells treated with prolactin/IFN-γ/TNF-α. Analysis of caspase-3 activation revealed that levels of cleaved caspase-3 were enhanced by approximately 1.8-fold in cells treated with IFN-γ/TNF-α in the absence or presence of prolactin (Figure 4E). In addition, RNA-sequencing results showed significant upregulation of several caspases and gasdermin D following IFN-γ/TNF-α treatment (Supplementary Data, Table S1). These findings indicate that combined treatment with IFN-γ and TNF-α promotes cell death.

### 3.5. Inhibition of iNOS Partially Reverses the Detrimental Effects of IFN-γ/TNF-α on β-Casein Expression and Acinar Morphology

**Figure 5:**
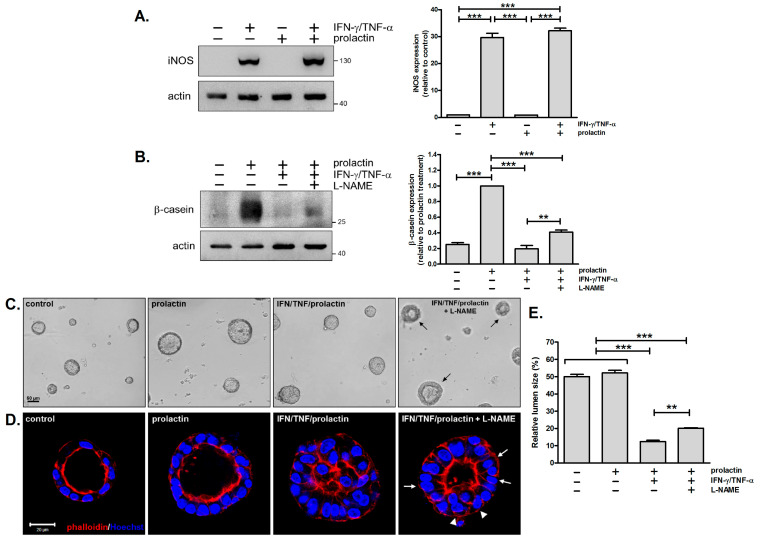
Inhibition of iNOS partially reverses the detrimental effects of IFN-γ/TNF-α on β-casein expression and acinar morphology. (**A**) Immunoblot analysis and quantification of iNOS expression (*n* = 3). *** *p* < 0.005. (**B**–**D**) Mammary cells were pretreated with IFN-γ/TNF-α in the absence or presence of the NOS inhibitor L-NAME (1 mM) for 1 h and then stimulated with prolactin for 2 days. (**B**) Immunoblot analysis and quantification of β-casein expression (*n* = 3). ** *p* < 0.01, *** *p* < 0.005. (**C**) Bright-field microscopy images show thicker outer rings in acini treated with cytokines/prolactin/L-NAME (arrows) (*n* = 3). Scale bar, 50 μm. (**D**) Confocal images of acini stained with rhodamine phalloidin (red) and Hoechst 33342 (blue). Arrows indicate cells displaying a columnar shape; arrowheads indicate regions with more than one cell layer (*n* = 3). Scale bar, 20 μm. (**E**) Relative lumen size in (**D**). ** *p* < 0.01, *** *p* < 0.005.

It has been reported that IFN-γ and TNF-α synergize to trigger inflammatory cell death through the STAT1/IRF1/iNOS/NO pathway [29]. From our RNA-sequencing analysis, we observed an upregulation of the *Nos2* gene in response to IFN-γ/TNF-α stimulation in mammary cells (Supplementary Data, Table S1). This was further confirmed by immunoblotting analysis, showing the robust induction of iNOS following combined cytokine treatment (Figure 5A). To investigate whether iNOS contributes to the detrimental effects of IFN-γ and TNF-α on β-casein expression and acinar morphology, a NOS inhibitor, L-NAME, was employed. It caused a two-fold increase in β-casein levels compared to cells treated with cytokines and prolactin in the absence of the inhibitor (Figure 5B, lane 3 vs. lane 4). However, L-NAME could not completely alleviate the inhibitory effect of the cytokines to fully restore β-casein expression (Figure 5B, lane 2 vs. lane 4).

We also examined the effect of L-NAME on acinar morphology. In acini treated with cytokines and prolactin, the application of L-NAME led to the appearance of outer rings. These rings were thick (Figure 5C, arrows), implying a thicker cell layer or multiple layers of cells surrounding the lumens. Examination of the 3D structure of the acini supported these results. For acini exposed to L-NAME, the lumens were expanded 1.6-fold (Figure 5E), but the cells retained their columnar shape (Figure 5D, arrows), with multiple layers of cells observed in certain regions (Figure 5D, arrowheads). Similar results were obtained with another iNOS inhibitor, 1400W (Supplementary Data, Figure S3). Collectively, combined treatment with IFN-γ and TNF-α induces iNOS expression, and inhibition of iNOS with L-NAME partially reverses the adverse effects of these cytokines on β-casein expression and acinar morphology.

## 4. Discussion

The immune system not only provides protection against intruding microorganisms and malignant host cells but also participates in tissue development and organogenesis [30]. Previous studies have shown that Th2 cytokines facilitate mammary gland development during pregnancy [15]. Here, we demonstrated that the Th1 cytokines IFN-γ and TNF-α inhibit milk protein synthesis and alter the structural organization of mammary acini. These adverse effects were partially restored by the inhibition of iNOS, which is induced by combined cytokine treatment. Our findings suggest that a Th1-skewed cytokine milieu, as observed during mastitis, compromises the architecture and function of mammary glands, contributing to lactation failure.

The 3D culture system employed here is physiologically relevant, given the use of primary mammary cells and the basement membrane-like matrix (Matrigel) to which normal epithelia adhere in vivo. These cells form 3D spheres with hollow lumens, reminiscent of alveoli in mammary glands. They are also capable of synthesizing milk in response to lactogenic hormones (prolactin, insulin, and hydrocortisone) [22,28]. Here, we observed impaired acinar structures characterized by reduced lumen size and disorganized cell arrangement following combined treatment with IFN-γ and TNF-α. These structural features could not be detected by conventional 2D cultures, highlighting the advantage of the 3D model. Interestingly, a disordered structure with a narrowed lumen would thwart milk drainage, thereby exacerbating milk stasis and further amplifying inflammation [6,17].

### 4.1. Detrimental Effects of IFN-γ/TNF-α on Milk Protein Expression

It has been reported that breast milk from women with subclinical mastitis exhibits a predominant Th1/proinflammatory cytokine profile, including TNF-α, IL-6, IL-8, IL-17, RANTES, IL-12p40/70, IFN-α, IFN-γ, CXCL-9, IP-10, MIP-1α, and MIP-1β [18]. IFN-γ and TNF-α, along with other cytokines, have been detected in breast milk from women with clinical and subclinical mastitis [31]. In a murine acute mastitis model, levels of TNF-α were increased in mammary glands after a lipopolysaccharide challenge, and transcriptome analysis revealed the upregulation of *Nos2* and genes involved in the IFN-γ response and TNF-α signaling [32]. Therefore, the use of IFN-γ and TNF-α in our study might simulate inflammatory conditions during mastitis. Consistent with the lactation insufficiency observed in mastitis, we found that IFN-γ and TNF-α inhibit prolactin-induced STAT5 tyrosine phosphorylation and β-casein expression (Figure 1). These results are similar to other findings reported earlier. IFN-γ has been demonstrated to downregulate β-casein and milk fat synthesis via the induction of autophagy [26], while TNF-α has been found to inhibit β-casein expression through the antagonism of STAT5 tyrosine phosphorylation by NF-κB [33]. We suspect another possibility involving the cytokine induction of the suppressor of cytokine signaling (SOCS) proteins, which counteract the JAK-STAT pathway. IFN-γ has been shown to upregulate SOCS-1, SOCS-2, SOCS-3, and CIS, and the overexpression of these SOCS proteins suppresses β-casein synthesis in mammary epithelial cells [34,35]. Of interest, SOCS1 deficiency leads to augmented STAT5 tyrosine phosphorylation and milk protein synthesis, indicating a negative impact of SOCS1 on prolactin signaling [35]. Our RNA-sequencing data showed significant inductions of *Socs-1* and *Cis* following combined cytokine treatment (Supplementary Data, Table S1). These results suggested the potential involvement of SOCS proteins in the inhibitory effect of cytokines on prolactin signaling and β-casein expression, which warrants further investigation.

### 4.2. Detrimental Effects of IFN-γ/TNF-α on Acinar Morphology

Alveologenesis is controlled by systemic hormones such as prolactin and progesterone, as well as local factors including insulin-like growth factor-II (IGF-II), the receptor activator of NF-κB ligand (RANKL), and heregulin [36]. Prolactin is especially important since it also governs luminal cell fate determination and promotes lactogenic differentiation through STAT5 [37]. In contrast, the Th1 cytokines IFN-γ and TNF-α not only inhibit milk protein expression but also disrupt acinar structures, as observed in this study (Figure 2). The influences of IFN-γ and TNF-α on cell morphology in 2D cultures have been documented. In response to IFN-γ, MCF-7 and MDA-MB231 cells become more elongated [38]. Similar results have been observed in various cell types following TNF-α or IFN-γ/TNF-α treatment [39,40,41]. However, our 3D culture model revealed additional structural abnormalities. We discovered that IFN-γ/TNF-α treatment leads to cell shape changes. Individual mammary cells within acini exhibited a columnar shape instead of their typical cuboidal form. Moreover, the 3D structure of acini was altered, resulting in small or no lumens, irregular arrangements of cells, and multiple cell layers in certain regions (Figure 2B). Interestingly, these aberrant features resemble the phenotype of *Stat5*-null mammary glands, consistent with our results that combined treatment with IFN-γ/TNF-α inhibits prolactin-induced STAT5 tyrosine phosphorylation (Figure 1B) [42]. Furthermore, given the role of STAT5 in specifying luminal cell fate, these cytokines may impair acinar structures by inhibiting luminal differentiation [37]. This notion is supported by a previous finding that IFN-γ downregulates the expression of mature luminal and luminal progenitor markers [7]. Likewise, *Stat1* ablation has been shown to sustain prolactin signaling and expand the CD61^+^ luminal progenitor population [43]. Our RNA-sequencing results are consistent with these findings, showing a decreasing trend in the expression of *Stat5a*, as well as mature luminal markers (progesterone receptor, GATA3, mucin 1) and luminal progenitor markers (CD61) (Supplementary Data, Table S1).

Similar to our results, mutation of the viral sensor 2′-5′-oligoadenylate synthetase 2 (*Oas2*) has been shown to cause lactation failure, accompanied by enhanced cell death, decreased cell proliferation, and vigorous IFN responses. In particular, phospho-STAT1 staining is detected in regions with packed and unexpanded alveoli [44]. This suggests an association between IFN signaling and aberrant alveolar morphology. Another study reported that the width of terminal end buds in mammary glands decreases during inflammation, reinforcing our findings that proinflammatory cytokines alter mammary gland structures [45].

In this study, we showed that while IFN-γ/TNF-α altered the acinar structure, apico-basolateral polarity was roughly preserved (Figure 3). These results are similar to observations in *Stat5*-null and *Prlr* (prolactin receptor)-null mammary glands [42]. Tight junctions are fundamental to establishing tissue polarity and controlling barrier permeability. By altering the expression and localization of junctional proteins, IFN-γ and TNF-α have been shown to compromise barrier function in intestinal and renal epithelium [46,47]. Disruption of the blood–milk barrier is also observed in mastitis, though IL-1β seems to be the key cytokine driving this adverse effect [27].

### 4.3. Involvement of iNOS in the Detrimental Effects of IFN-γ/TNF-α

Combined treatment with IFN-γ and TNF-α promotes cell death under various conditions. In diseases associated with cytokine storms, such as SARS-CoV-2 infection and cytokine shock syndromes, IFN-γ and TNF-α synergize to induce inflammatory cell death via the STAT1/IRF1/iNOS/NO pathway, leading to tissue damage and mortality [29]. IFN-γ and TNF-α are also the key effector cytokines in anti-tumor immunity; one mechanism is ascribed to their role in triggering cell death [20,48,49]. Interestingly, a recent study has discovered that CD4 Th1 cells, along with myeloid cells, can exert a cytolytic effect on tumor cells via IFN-γ and TNF-α [20]. We also observed that combined treatment with IFN-γ and TNF-α causes cell death in mammary acini, although the extent is less severe (Figure 4B). This might be due to the lower cytokine concentration used here (10 ng/mL) compared to other studies (20 ng/mL~160 ng/mL) [20,29,49]. Additionally, IFN-γ/TNF-α induced iNOS expression, and the inhibition of iNOS partially restored acinar morphology and β-casein expression (Figure 5). These results suggested that iNOS plays a part in IFN-γ/TNF-α-mediated effects, but other mechanisms might also be involved. One possibility is the induction of cellular senescence by IFN-γ/TNF-α [50,51,52,53]. We evaluated the expression of p16INK4a, a marker of senescence, and found no induction following cytokine treatment. However, the potential involvement of senescence cannot be entirely excluded at this stage.

### 4.4. Implications for Mastitis

Mastitis commonly occurs in women who are breastfeeding, with clinical manifestations including reduced milk secretion, breast pain, and even the systemic symptom of fever. Diagnosis is usually made based on the presence of clinical symptoms. Examination of the immune profiles of the serum and breast milk has been reported, and the emergence of a proinflammatory signature before or during disease onset may offer a means for early prediction and detection [18,31]. Using 3D cultures of primary mouse mammary cells, we discovered that IFN-γ and TNF-α together inhibit milk protein expression and disrupt acinar architecture. These cytokines have been detected in women with mastitis, and their effects are similar to the lactation insufficiency and tissue damage observed in human mastitis, thereby supporting the translational relevance of our study.

The etiology of mastitis is multifactorial, but infection and milk stasis are considered the primary causes [16,17]. During lactation, infection can shift the tolerant immune environment toward a Th1-dominant state through activation of the adaptive responses [54]. Consequently, CD4^+^ Th1 cells produce both IFN-γ and TNF-α, while CD8^+^ T cells contribute to additional IFN-γ, and myeloid cells such as macrophages serve as sources of TNF-α. However, milk stasis triggers a proinflammatory response by activating the innate immune pathways. Mechanical stress generated by milk accumulation can damage cells, causing the release of intracellular content, termed danger signals. These activate pattern recognition receptors, such as Toll-like receptors (TLRs) or NOD-like receptors (NLRs), leading to NF-κB-mediated TNF-α expression [6,17]. Mitochondrial damage may occur under conditions of cellular stress, and the released mitochondrial DNA can activate the cyclic GAMP synthase (cGAS)-stimulator of the IFN gene (STING) pathway, resulting in the production of type I IFNs [6,55]. Although type I IFNs are different from IFN-γ, they share some signaling pathways, such as JAK/STAT. Thus, IFN and TNF-α may both contribute to the inflammatory responses associated with mastitis, whether initiated by infection or milk stasis.

Although our in vitro system provides a means to dissect cytokine effects on mammary epithelia, it cannot fully recapitulate mastitis in vivo due to the absence of other immune cells and mediators. Therefore, while our findings provide mechanistic insight, further studies with co-cultures of mammary cells and immune cells or animal models are needed [56].

## 5. Conclusions

In summary, we demonstrated that combined treatment with IFN-γ and TNF-α inhibits prolactin signaling and subsequent milk protein expression, accompanied by the disruption of acinar architecture. This cytokine treatment also induces iNOS expression, while the inhibition of iNOS improves both milk protein synthesis and acinar structures. These findings suggest that in conditions of Th1 skewing, such as mastitis, IFN-γ and TNF-α play key roles in lactation insufficiency and tissue damage. Targeting the iNOS pathway may thus be a therapeutic strategy for mastitis and other inflammation-associated disorders.

## Figures and Tables

**Table 1:** Antibodies used in immunoblotting and immunofluorescence analyses.

Antibody	Source	Concentration
β-casein	Santa Cruz Biotechnology, sc-17971 (Santa Cruz, CA, USA)	0.4 μg/mL
phospho-STAT5	Millipore, #05-495 (Temecula, CA, USA)	1.5 μg/mL
STAT5	Santa Cruz Biotechnology, sc-835 (Santa Cruz, CA, USA)	0.6 μg/mL
iNOS	Santa Cruz Biotechnology, sc-650 (Santa Cruz, CA, USA)	1.0 μg/mL
cleaved caspase-3	Cell Signaling Biotechnology, #9661 (Beverly, MA, USA)	1:500
actin	Sigma-Aldrich, #A-5060 (St. Louis, MO, USA)	1:1000
ZO-1	Invitrogen, #40-2200 (Camarillo, CA, USA)	1:100
β-catenin	BD Biosciences, #610153 (San Jose, CA, USA)	1:200

## Data Availability

The data presented in this study are available on request from the corresponding author.

## Supplementary Materials

The following supporting information can be downloaded at: https://www.mdpi.com/article/10.3390/biomedicines13061455/s1, Figure S1: Combined treatment with IFN-γ and TNF-α reduces the lumen size of mammary acini; Figure S2: Combined treatment with IFN-γ and TNF-α leads to disorganized structures, with no lumen in some mammary acini; Figure S3: Inhibition of iNOS by 1400 W partially reverses the detrimental effect of IFN-γ/TNF-α on β-casein expression and acinar morphology; Table S1: RNA-sequencing results.

## Abbreviations

The following abbreviations are used in this manuscript:

IFN-γ	Interferon-γ
IL	Interleukin
TNF-α	Tumor necrosis factor-α
Th	T helper
