# Supplementary material for: Th1 Cytokines Inhibit Acinar Morphogenesis and Milk Protein Expression in 3D Mammary Cultures
Source: Biomedicines. 2025 Jun 12;13(6):1455. doi: 10.3390/biomedicines13061455 (PMC12191074; doi:10.3390/biomedicines13061455)
Supplement: Supplementary file 1 [file biomedicines-13-01455-s001.zip › biomedicines-3589344-supplementary.pdf]

Supplementary Data

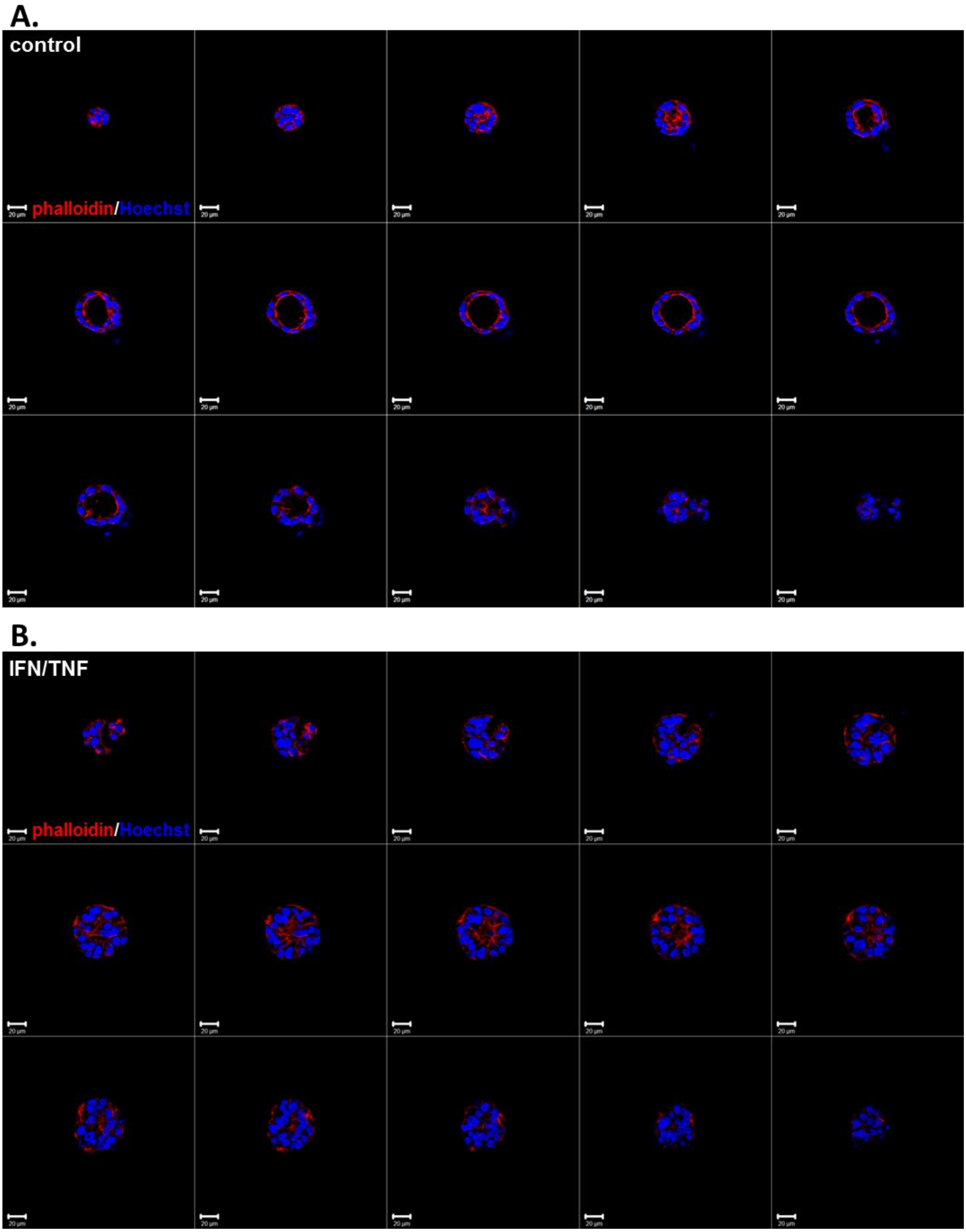

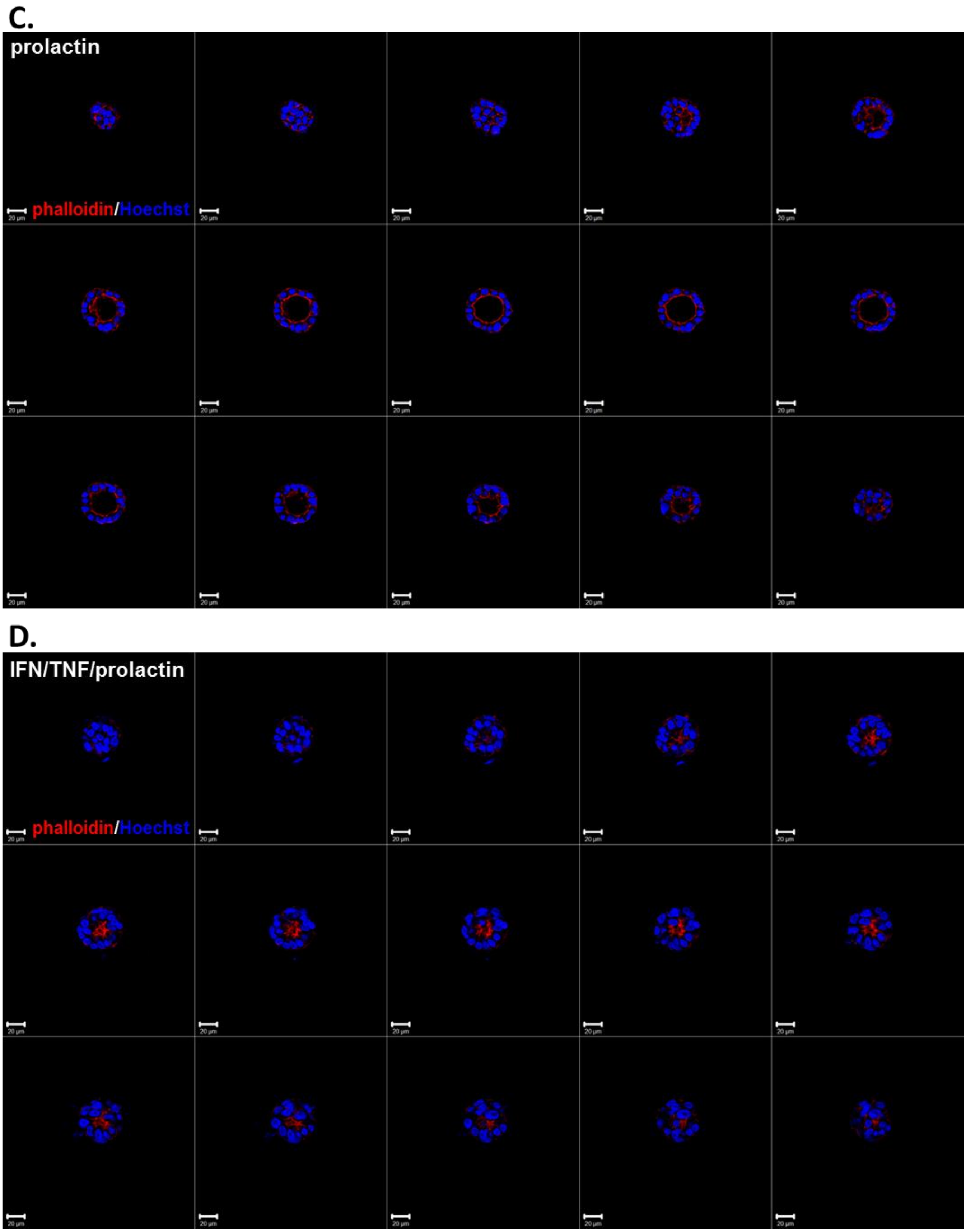

Figure S1. Combined treatment with IFN-γ and TNF-α reduces the lumen size of mammary acini. Mammary cells cultured on Matrigel were either untreated (**A**), treated with IFN-γ/TNF-α for 2 d (**B**), treated with prolactin for 2 d (**C**), or pretreated with IFN-γ/TNF-α for 1 h followed by stimulation with prolactin for 2 d (**D**). Cells were stained with rhodamine-phalloidin (red) and Hoechst 33342 (blue) and subjected to confocal microscopy. A series of images were taken from the Z-axis of acini. Scale bar, 20 μm.

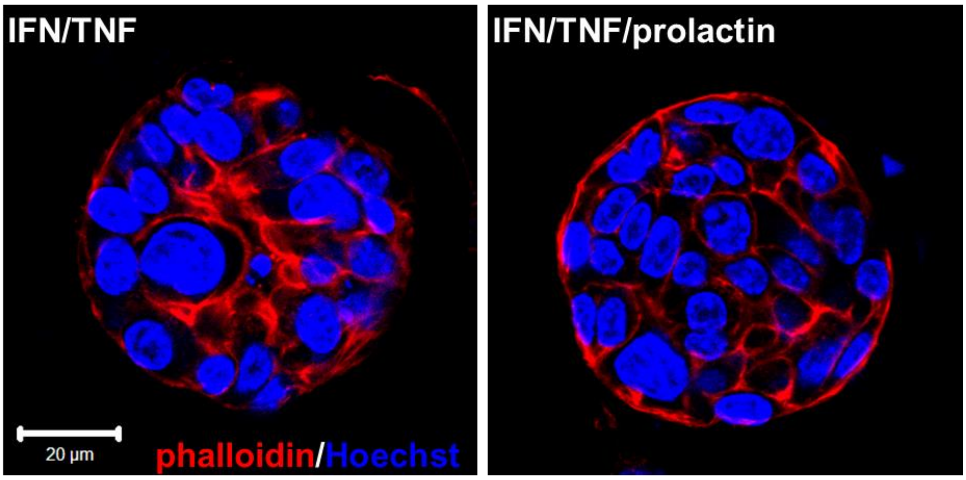

Figure S2. Combined treatment with IFN-γ and TNF-α leads to disorganized structure with no lumen in some mammary acini. Mammary cells cultured on Matrigel were treated with IFN-γ/TNF-α for 2 d or pretreated with IFN-γ/TNF-α for 1 h followed by stimulation with prolactin for 2 d. Cells were stained with rhodamine-phalloidin (red) and Hoechst 33342 (blue) and subjected to confocal microscopy. Images were taken from the center of acini. Scale bar, 20 μm.

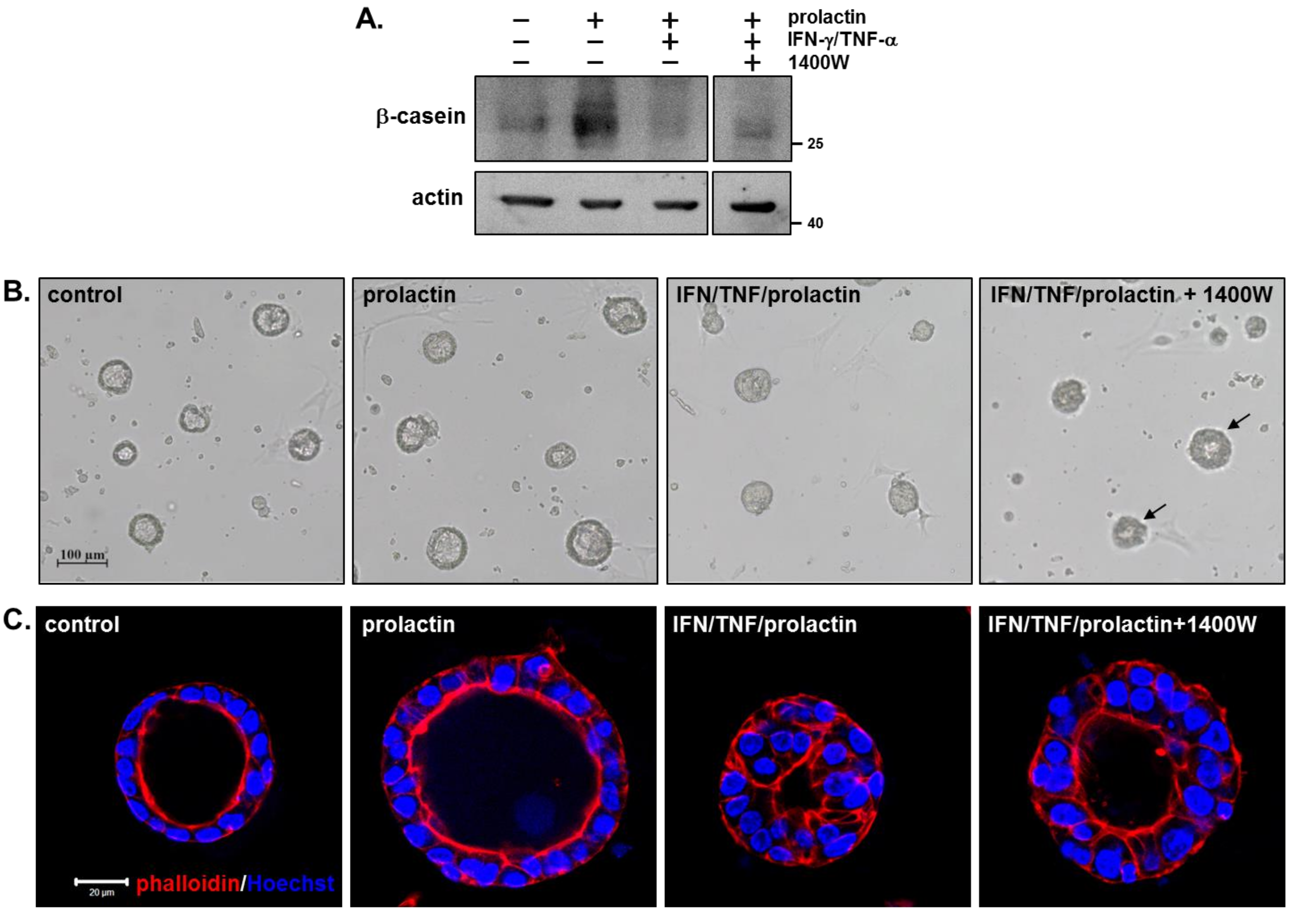

Figure S3. Inhibition of iNOS by 1400W partially reverses the detrimental effect of IFN-γ/TNF-α on β-casein expression and acinar morphology. Mammary cells cultured on Matrigel were pretreated with IFN-γ/TNF-α in the absence or presence of the iNOS inhibitor 1400W (100 μM) for 1 h and then stimulated with prolactin for 2 d. (**A**) Total cell lysates were analyzed by immunoblotting with antibodies to β-casein and actin. Actin was used as a loading control. (**B**) Photographs were taken under a bright-field light microscope. Thicker outer rings were detected in acini treated with cytokines/prolactin/1400W (arrows). Scale bar, 100 μm. (**C**) Cells were stained with rhodamine-phalloidin (red) and Hoechst 33342 (blue) and subjected to confocal microscopy. Images were taken from the center of acini. In cytokines/prolactin/1400W-treated acini, the lumen was expanded. Scale bar, 20 μm.

Category	Gene	FPKM	
		control	IFN-γ/TNF-α
	Nos2 (iNOS)	3.36	748.37
Cell Death	caspase 1	42.56	163.88
	caspase 4	10.02	127.57
	caspase 7	19.51	115.33
	caspase 12	25.96	159.90
	gasdermin D	2.37	18.18
STAT Pathway	SOCS1	0.54	70.58
	CIS	5.00	26.54
	STAT5a	78.49	49.33
Mature Luminal Markers	progesterone receptor	0.89	0.18
	GATA3	180.98	92.89
	mucin 1	259.26	147.70
Luminal Progenitor Markers	CD14	502.72	375.74
	integrin alpha2 (CD49b)	26.18	33.41
	integrin beta3 (CD61)	75.45	7.69

Table S1. RNA-sequencing results. Mammary cells cultured on Matrigel were untreated or treated with IFN-γ/TNF-α for 16 h. Total RNA was subjected RNA-seq analysis.
